# Comparative effectiveness of levofloxacin versus third-generation cephalosporins and predictors of treatment outcomes in upper respiratory tract scleroma: a tertiary center experience

**DOI:** 10.1038/s41598-026-66683-y

**Published:** 2026-08-20

**Authors:** Ahmed Ibrahim Khaleel, Rabeh Khairy Saleh, Chrestina Monir Fekry, Fatma Wafik Shahin

**Affiliations:** 1 Otorhinolaryngology Department, Faculty of Medicine, Minia university, Minia, Egypt; 2 Pathology Department, Faculty of Medicine, Minia University, Minia, 61657 Egypt; 3 Public health and preventive medicine Department, Faculty of Medicine, Minia University, Minia, Egypt; 4 Unit of Phoniatrics, Otorhinolaryngology Department, Faculty of Medicine, Minia University, Minia, Egypt

**Keywords:** Rhinoscleroma, Upper respiratory tract scleroma, Levofloxacin, Third-generation cephalosporins, Diseases, Health care, Medical research

## Abstract

Upper respiratory tract scleroma is a chronic granulomatous disease that primarily affects the nose but may involve any part of the upper respiratory tract. The mainstay of treatment is prolonged antibiotic therapy. This prospective comparative study included 90 adults with upper respiratory tract scleroma treated at Minia University Hospital, Egypt, between September 2024 and August 2025. Patients were randomly allocated into two equal groups using closed sealed opaque envelopes to receive levofloxacin or third-generation cephalosporins for three months. The mean age was 36.3 ± 10.4 years, and 90% were females. Nasal obstruction (96.7%) and nasal crustation (90%) were the most common manifestations. Rhinolaryngoscleroma was diagnosed in 86.7% of patients, whereas 13.3% had rhinoscleroma. Clinical improvement was significantly higher with levofloxacin than with third-generation cephalosporins (80% vs. 60%, *P* = 0.038). In the multivariable analysis, treatment type and pharyngeal involvement remained significant predictors of clinical improvement. Levofloxacin treatment was associated with higher odds of improvement (OR = 5.8, *P* = 0.015). These findings highlight the importance of early diagnosis and appropriate antibiotic selection in the management of upper respiratory tract scleroma.

**Supplementary Information:**

The online version contains supplementary material available at 10.1038/s41598-026-66683-y.

## Introduction

Upper respiratory tract scleroma is a chronic specific inflammation that primarily affects the nasal cavity (rhinoscleroma) but may also affect any part of the respiratory tract including pharynx, larynx, and trachea and is caused by *Klebsiella rhinoscleromatis* (KR) bacilli^1,2^.

The disease is endemic in Egypt^3^, as well as other isolated regions throughout the world, such as Chile, Indonesia, India, Central Africa, Central America, and other Middle Eastern nations^4^.

Rhinoscleroma (RS) is more common in rural areas with low socioeconomic status. Crowding, inadequate nutrition, and poor hygiene all contribute to the disease’s spread. Women are impacted more often than men^2^.

The characteristic histopathological features of scleroma are observed primarily during the granulomatous phase. This stage showed heavy inflammatory cellular infiltration formed mainly of lymphocytes and plasma cells, along with Russell bodies and the pathognomonic Mikulicz cells which are large foamy macrophages filled with KR bacilli. Mikulicz cells are typically absent or scarce during the early catarrhal and late sclerotic phases, but they are most prominent in the intermediate proliferative phase. In contrast, the fibrotic phase is characterized by extensive collagen deposition and minimal inflammatory cell infiltration^5–8^. The intra-cytoplasmic bacilli are best visualized using special stains such as periodic acid–Schiff (PAS) or Giemsa^5^.

Since the KR bacilli are an intracellular organism, they are resistant to many antibiotics and won’t be exposed to high drug concentrations. Because fibrosis interferes with the normal function of the respiratory passages, a therapeutic treatment is challenging^9^.

There is no established treatment for upper respiratory tract scleroma. The majority of treatment is medical by antibiotics such as doxycycline/tetracycline, rifampicin, ciprofloxacin, and trimethoprim/sulfamethoxazole, either alone or in combination and the ideal length of treatment has not been conclusively determined with satisfactory outcomes after six weeks to six months^10^.

This fact inspired us to look for the best antibiotic to treat upper respiratory tract scleroma while minimizing adverse effects. Here, we examined the results of a case series of patients treated with levofloxacin as compared to third-generation cephalosporins.

### Aim of the work

This study aimed to evaluate and compare the therapeutic efficacy of levofloxacin versus third-generation cephalosporins in the management of upper respiratory tract scleroma.

### Patients and methods

This was a prospective comparative study conducted at Otorhinolaryngology and phoniatric clinics, Faculty of Medicine, Minia University Hospital, Egypt, during the period between September 2024 and August 2025, comprising 90 adult patients in our highly endemic area who were diagnosed with upper respiratory tract scleroma by clinical diagnosis based on typical disease manifestations and the presence of the typical histopathological picture of upper respiratory tract scleroma. The study objectives and procedures were thoroughly explained to all participants, and written informed consent was obtained from each participant prior to study enrollment.

The study was submitted for review and received approval from the Medical Research Ethics Committee of the Faculty of Medicine, Minia University (approval number: 1272/9/2024, approval date: 9/9/2024), in accordance with the ethical principles outlined in the Declaration of Helsinki.

### Inclusion criteria

Patients aged 18–60 years with clinically and histopathologically confirmed upper respiratory tract scleroma were included.

### Exclusion criteria

Patients who had previous surgical treatment for upper respiratory tract scleroma, aged less than 18 or older than 60 years, chronic kidney or liver disease, pregnant or lactating women, in the inactive stage of the disease, and those who did not complete their follow-up visits (dropped out).

### Sample size calculation

The sample size was calculated using G*Power software (version 3.1.9.4) for comparing two independent proportions. Based on treatment outcomes reported in previous studies on rhinoscleroma, a moderate-to-large effect size (Cohen’s h = 0.60) was assumed according to Cohen’s recommendations^11^. Using a two-sided significance level (α = 0.05) and a statistical power of 80%, the minimum required sample size was calculated to be 90 patients (45 patients per group).

Eligible participants were randomly allocated into two equal treatment groups using the sealed opaque envelope method. Treatment allocation was concealed in sealed opaque envelopes prepared before patient enrollment and opened only after each participant had been enrolled.

### Patients were subjected to the following

1. Complete medical history, with special attention to contact with sick family members, the duration of complaint, past medication use, prior head and neck surgeries, common immunodeficiency causes like HIV, diabetes mellitus (DM), or immunosuppressive treatment history.
2. Complete clinical otorhinolaryngological examination.
3. Punch biopsy from the nasal granulations, or any suspected unhealthy lesions in the nose, nasopharynx, or oropharynx under local anesthesia, and any suspected unhealthy lesions in the larynx were taken under general anesthesia. Biopsies were processed for histopathological examination.
4. The allocated treatment regimen was as follows:

- Group 1 (G1): received levofloxacin.
- Group 2 (G2): received third-generation cephalosporins.

To minimize potential sources of bias, all participants were assessed using standardized clinical and histopathological criteria. All participants underwent the same follow-up schedule and outcome assessment throughout the study period.

### Histopathological and immunohistochemical procedures

As part of the standard histopathology procedure, tissue biopsies were preserved in 10% neutral buffered formalin. Tissue sections were processed, embedded in paraffin wax, cut at a thickness of 3 μm using a microtome, and stained with hematoxylin and eosin (H&E), Giemsa, PAS, and Masson trichrome to determine the histological details^12^.

The staining process was carried out at room temperature after being deparaffinized in xylene, the tissue pieces were rehydrated in reduced alcohol and rinsed for 2 min in distilled water. Tissues were treated in periodic acid for 10 min, rinsed with distilled water, then incubated in Schiff reagent for 15 min before being differentiated in two sodium metabisulphite changes for PAS staining. Following two rounds of distilled water washing, Mayer hematoxylin was used as a counterstain. For Giemsa stain, sections were incubated in a working Giemsa solution for 20 min, followed by a distilled water wash and differentiation in 0.5% acetic acid. After being dehydrated in upgraded alcohol, each section was cleaned in xylene and mounted as usual^13–15^.

The state of the covering epithelium, the intense of inflammatory response, the type and relative dominance of inflammatory cells, and the degree of fibrosis were among the specific histological findings that were assessed^3^.

Five-micrometer-thick sections were prepared from the specimens and immunohistochemistry (IHC) was performed for anti-CD68 antibody (Abcam, ab192847, 1:100) according to the standard IHC protocol^16^.

An Olympus BX50 microscope was used in the current study to characterize histopathological alterations at magnifications of 10×, 200×, 400×, and 1000x. The 1000x magnification was performed using the oil-immersion technique. A light microscope that was attached to a camera was used to capture the images.

### Treatment

Antibiotics were administered for 3 months, with follow-up for the clinical outcomes and cure of the disease.

Clinical improvement was defined as the resolution or improvement of the presenting symptoms and clinical signs after treatment, based on clinical and endoscopic findings during follow-up.

### Statistical analysis

Data were analyzed using IBM SPSS Statistics software version 22 (IBM Corp., Armonk, NY, USA). Continuous variables were expressed as mean ± standard deviation (SD), while categorical variables were presented as frequencies and percentages. Comparisons between categorical variables were performed using the Chi-square test. A p-value < 0.05 was considered statistically significant for all statistical tests. Univariate binary logistic regression analysis was initially performed to identify potential predictors of clinical improvement. Variables with a p-value < 0.05 in the univariate analysis were entered into a stepwise multivariable binary logistic regression model to identify independent predictors of clinical improvement. The final model was selected based on the stepwise selection procedure. Adjusted odds ratios (ORs) with 95% confidence intervals (CIs) were reported. Only participants who completed the follow-up period were included in the final analysis; therefore, no missing outcome data were analyzed. Figures were prepared using Microsoft Excel 2010.

## Results

### Sociodemographic characteristics of the studied case

**Table 1:** Sociodemographic characteristics of studied case (n=90)

Variable	Value
Age	36.3 ± 10.4
Mean ± SD	(18–52)
Range	
Gender n (%)	9 (10%)
Male	81 (90%)
Female	
Occupation n (%)	9 (10%)
Student	66 (73.3%)
Housewife	6 (6.7%)
Farmer	3 (3.3%)
Worker	6 (6.7%)
Teacher	
Smoking	6 (6.7%)
Yes	84 (93.3%)
No	
Contact with diseased relative	9 (10%)
Yes	81(90%)
No	
Duration of complaint (months)	18.87 ± 8.8
Mean ± SD	(6–36)
Range	
History of Chronic diseases	4 (4.4%)
Diabetes	7 (7.8%)
Hypertension	

SD=Standard Deviation, n= number.

Table 1 shows that the mean age of the studied cases was 36.3±10.4 years, ranging from 18 to 52 years. Most of the cases (90%) were females. Most cases (93.3%) were non-smokers, 90% had no family history of upper respiratory tract scleroma. Mean duration of complaint was 18.87±8.8 months.

### Frequencies of clinical manifestations among studied cases

**Fig. 1:**
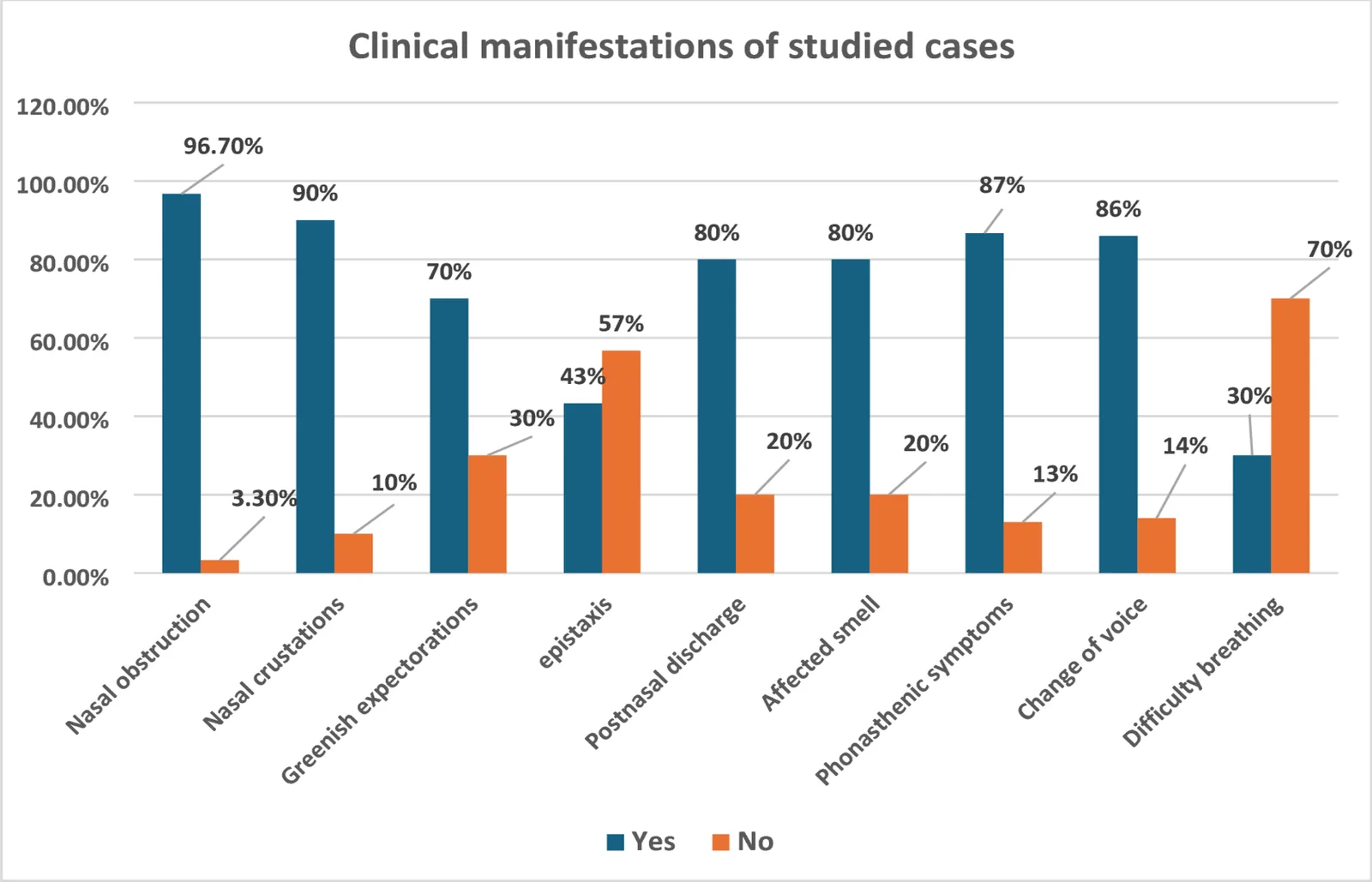
Frequencies of clinical manifestations among studied cases.

In Fig. 1, regarding clinical symptoms, the most common complaint was nasal obstruction (96.7%). Followed by nasal crustations (90%). Phonasthenic symptoms were revealed among 87% of cases. Change of voice was found among 86% of cases. Postnasal discharge was found in 80% of cases, and 70% had expectoration of greenish discoloration. Affected smell was found in 80% of cases.

### Signs of studied cases

**Table 2:** Signs of studied cases (n = 90).

Variable	Value
Affected pharynx	66 (73.3%)
Affected	24 (26.7%)
Not affected	
Dysphonia grade (%)	15 (16.7%)
0	39 (43.3%)
1	36 (40%)
1–2	
VF color	12 (13.3%)
Normal	60 (66.7%)
Mild congestion	9 (10%)
Moderate congestion	9 (10%)
Severe congestion	
VF mucosa	12 (13.3%)
Normal mucosa	36 (40%)
Unhealthy mucosa	33 (36.7%)
Irregular mucosa	9 (10%)
Hypertrophied	
VF mobility	87 (96.7%)
Mobile	3 (3.3%)
Both restricted in abduction	
Supraglottic region	69 (76.7%)
Normal	3 (3.3%)
Sticky yellow secretion	9 (10%)
Irregular mucosa	9 (10%)
Ventricular fold hypertrophy	
Subglottic region	12 (13.3%)
Normal	60 (66.7%)
Greenish crustations	18 (20%)
Subglottic stenosis with greenish crustations	
Inter-arytenoid region	30 (33.3%)
Normal	30 (33.3%)
Greenish crustations	18 (20%)
Greenish crustations and irregular mucosa	9 (10%)
Granulation tissue and greenish crustations	3(3.3%)
Irregular mucosa	
Stage	48 (53.3%)
Atrophic with roomy nose	42(46.7%)
Granulation	

VF= Vocal Fold, n= number.

Table 2 shows that grade 1 dysphonia was observed in 43.3% of cases. Approximately 66.7% had mildly congested vocal folds (VFs), while 40% had unhealthy VFs. Most cases (96.7%) had mobile VFs. Approximately 76.7% had no supraglottic involvement. Greenish crustations were observed in the subglottic region in 66.7% of cases. Nearly half of the cases (53.3%) were in the atrophic stage. Regarding the diagnostic type of scleroma, 86.7% had rhinolaryngoscleroma, whereas 13.3% had rhinoscleroma.

**Table 3:** Histopathological findings of upper respiratory tract scleroma cases.

Item	Value
Covering epithelium	72 (80%)
Squamous	9 (10%)
Shedded	9 (10%)
Columnar	
Inflammatory reaction	60 (66.7%)
Dense	30 (33.3%)
Moderate	
Russell bodies	60 (66.7%)
Yes	30 (33.3%)
No	
Dominant inflammatory cell	54 (60%)
Plasma cells	15 (16.7%)
Neutrophils	15 (16.7%)
Lymphocytes	6 (6.7%)
Eosinophils	
Fibrosis	60 (66.7%)
Dense	30 (33.3%)
Focal	

**Fig. 2:**
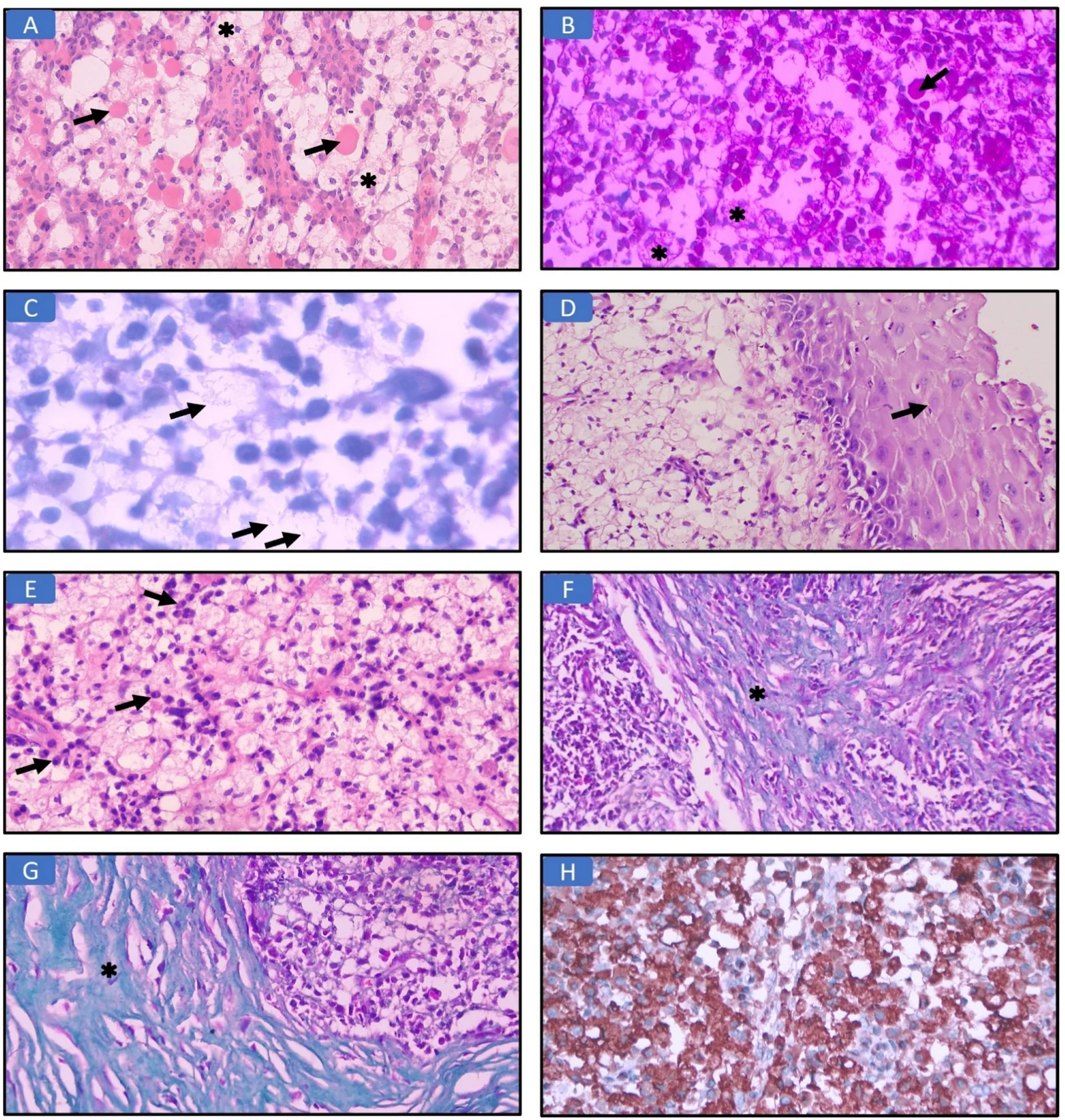
Histopathological and IHC findings in scleroma **(A)** Scleroma showed numerous Mikulicz cells (asterisks) and Russell bodies (arrows) (H&E, ×400). **(B)** Mikulicz cells (asterisks) showed vacuolated cytoplasm and Russell bodies (arrow) (PAS, ×400). **(C)** The Mikulicz cells showed *Klebsiella rhinoscleromatis* bacilli within the cytoplasm (arrows) (Giemsa, ×1000). **(D)** The picture shows squamous covering (arrow) and underlying Mikulicz cells of scleroma (H&E, ×400). **(E)** Scleroma showed predominant plasma cells (arrows) (H&E, ×400). **(F)** Scleroma with focal fibrosis (asterisk) (masson trichrome, ×400). **(G)** Scleroma with dense fibrosis (asterisk) (masson trichrome, ×400). **(H)** Diffuse deposits of CD68 within the cytoplasm of Mikulicz cells (IHC, ×400).

Table 3 and fig. 2 show the histopathological findings of upper respiratory tract scleroma cases. The most common covering epithelium was squamous (80%). A dense inflammatory reaction was observed in 66.7% of cases. Russell bodies were identified in 66.7% of cases, and 66.7% of cases showed dense fibrosis.

### Type of treatment of upper respiratory tract scleroma and improvement

**Table 4:** Type of treatment of upper respiratory tract scleroma and improvement.

	Levofloxacin	Third-generation cephalosporin	Significance
All cases			
Improved	36 (80%)	27 (60%)	χ2 = 4.29 *P* = 0.038 *
Not improved	9 (20%)	18 (40%)	
Total	45	45	

*χ2 = Chi-square test* * Significant level of p- value is < 0.05

The percentage of improvement was significantly higher among cases treated with levofloxacin (80%) compared to cases treated with third-generation cephalosporin (60%), (*P* = 0.038) (Table 4).

### Follow up clinical manifestations of patients

**Table 5:** Follow up clinical manifestations of patients (among cases received levofloxacin and third-generation cephalosporin).

Clinical manifestation	Treatment	No improvement	Partial improvement	Complete improvement	Significance
Nasal obstruction	Levofloxacin	5 (11.2%)	20 (44.4%)	20 (44.4%)	χ2 = 7.6 P= 0.03*
	Cephalosporin	8 (20.5%)	17 (43.6%)	14 (35.9%)	
Nasal crustations	Levofloxacin	6 (14.3%)	2 (4.8%)	34 (80.9%)	χ2 = 6.4 P = 0.04*
	Cephalosporin	14 (35.9%)	5 (12.8%)	20 (51.3%)	
Greenish expectoration	Levofloxacin	0(0%)	9 (26.4%)	25 (73.6%)	χ2 = 13.22 P = 0.001 *
	Cephalosporin	10 (26.3%)	6 (15.8%)	22 (57.9%)	
Smell	Levofloxacin	9 (25%)	3 (8.3%)	24 (66.7%)	χ2 = 8 P = 0.018*
	Cephalosporin	18 (50%)	6 (16.7%)	12 (33.3%)	
Epistaxis	Levofloxacin	0 (0%)	3(25%)	9 (75%)	χ2 = 6.1 P = 0.047*
	Cephalosporin	9 (37.5%)	3 (12.5%)	12 (50%)	
Post-nasal discharge	Levofloxacin	9 (25%)	6 (16.7%)	21(58.3%)	χ2 = 2.5 *P* = 0.29
	Cephalosporin	15 (41.7%)	6 (16.75)	15 (41.7%)	
Affected pharynx	Levofloxacin	0 (0%)	6 (20%)	24 (80%)	χ2 = 9.53 P = 0.009*
	Cephalosporin	9 (25%)	3 (8.3%)	24 (66.7%)	
Dysphonia	Levofloxacin	3 (7.1%)	10 (23.8%)	29 (69.1%)	χ2 = 13.56 P = 0.001*
	Cephalosporin	10 (28.6%)	2 (5.7%)	23(65.7%)	
VF color	Levofloxacin	0 (0%)	18 (42.9%)	24 (57.1%)	χ2 = 12.47 P = 0.002*
	Cephalosporin	9 (25%)	9 (25%)	18 (50%)	
VF mucosa	Levofloxacin	6 (14.3%)	0 (0%)	36 (85.7%)	χ2 = 5.57 *P* = 0.06
	Cephalosporin	9 (25%)	3 (8.3%)	24 (66.7%)	
Supraglottic structures	Levofloxacin	-	5 (50%)	5 (50%)	χ2 = 0.4 *P* = 0.53
	Cephalosporin	-	7 (63.6%)	4 (36.4%)	
Subglottic region	Levofloxacin	11 (28.2%)	5 (12.9%)	23 (58.9%)	χ2 = 1.29 *P* = 0.53
	Cephalosporin	14 (38.9%)	5 (13.9%)	17 (47.2%)	
Inter-artenoid region	Levofloxacin	3 (10%)	9 (30%)	18 (60%)	χ2 = 2.7 *P* = 0.26
	Cephalosporin	0 (0%)	9 (42.9%)	12(57.1%)	

*VF = Vocal Fold* *χ2 = Chi-square test* * Significant level of p- value is < 0.05

Complete improvement was more frequently observed with levofloxacin than with third-generation cephalosporins in most clinical manifestations, particularly nasal obstruction, nasal crustations, greenish expectoration, olfactory function, epistaxis, pharyngeal involvement, dysphonia, and vocal fold color, with statistically significant differences between the two treatment groups (*P* < 0.05). In contrast, no significant differences were observed between the two treatment groups regarding postnasal discharge, vocal fold mucosal involvement, supraglottic involvement, subglottic involvement, or interarytenoid involvement (*P* > 0.05). Overall, these findings suggest that levofloxacin was associated with better improvement in several, but not all, clinical manifestations (Table 5).

**Fig. 3:**
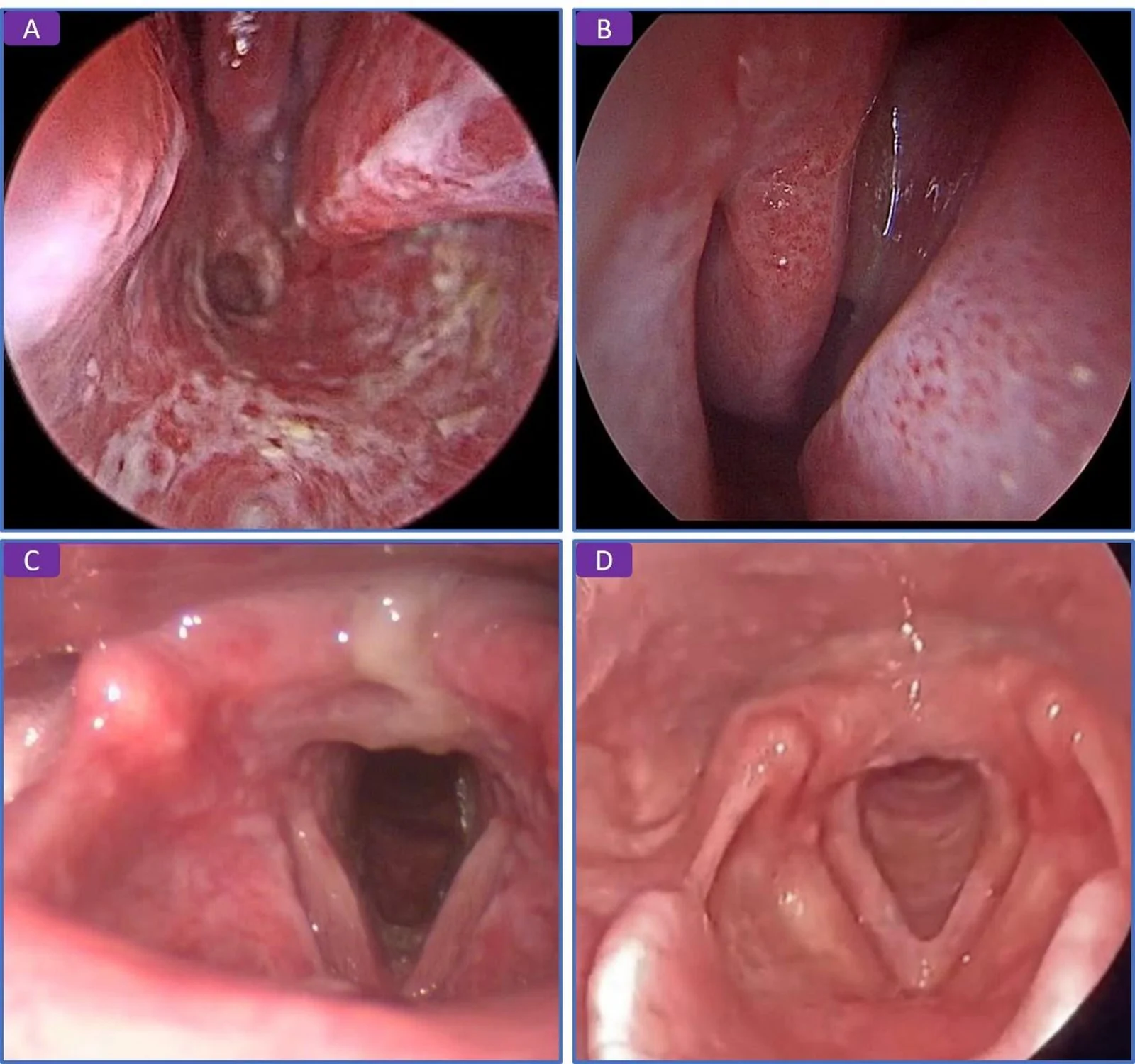
Endoscopic views of the left nasal cavity and laryngeal involvement before and after treatment. Endoscopic view of atrophic unhealthy mucosa in the left nasal cavity before treatment (**A**) and significant improvement after levofloxacin therapy (**B**). Laryngeal crustations before treatment (**C**) and significant improvement after levofloxacin therapy (**D**).

Representative endoscopic images (Fig. 3) are shown to illustrate the observed clinical improvement following treatment with levofloxacin.

### Factors predicting improvement among cases

**Table 6:** Factors predicting improvement among cases.

Item	Univariate regression			Multivariate regression		
	Odds ratio	CI	P value	Odds ratio	CI	P value
Age	0.99	0.95–1.04))	0.73			
Gender	Ref	Ref	Ref			
Male	0	(0-0.9)	0.99			
Female						
Occupation	Ref	Ref	Ref			
Student	0.1	(0-0.8)	0.86			
Housewife	0.01	(0-0.8)	0.99			
Farmer	0.1	(0-0.9)	0.99			
Worker	0.2	(0-0.9)	0.99			
Teacher						
Smoking	0.1	(0-0.9)	0.99			
Family history	0.84	(0.19–3.65)	0.82			
Complaint Duration	1.02	0.97–1.07))	0.54			
Nasal Obstruction	0.1	(0-0.9)	0.99			
Nasal crustations	0.1	0-0.9))	0.99			
Green Crustrations	1.75	(0.61–4.99)	0.3			
Smell	0.1	(0-0.9)	0.99			
Anosmia	0.1	(0-0.9)	0.99			
Hyponosmia						
Epistaxis	0.94	0.38–2.33))	0.89			
Stage	2.2	0.86–5.6))	0.1			
Postnasal Discharge	0.4	0.11–1.5))	0.18			
Not affected pharynx	3.4	1.27–9.1))	0.015*	0.12	0.02–0.75))	0.023*
Absence of dysphonia	14.68	4.47–48.28))	0.001*	0.6	0-0.7))	0.9
VF color Normal	9.29	2.59–33.3))	0.001*	0.1	(0–1)	0.9
Normal VF mucosa	5.98	2.57–13.9))	0.001*	0.7	(0-0.9)	0.8
Mobile VFs	0.1	0-0.9))	0.99			
No supraglottic region	2.53	1.09–5.8))	0.03*			
No Subglottic region	10.55	2.98–37.4))	0.001*			
Inter-arytenoid region	2.07	1.22–3.5))	0.007*	0.9	0-0.9))	0.81
(no irregular mucosa)						
Treatment (Levofloxacin)	2.67	1.04–6.85))	0.04*	5.8	1.4-24.02))	0.015*
Covering epithelium	6	(1.36–26.5)	0.018*			
Squamous	4	0.56–28.4))	0.17			
Shedded						
Inflammatory reaction	2.15	0.76–6.1))	0.15			
Russell bodies No	2	0.79–5.1))	0.15			
Dominant inflammation	0.5	0.13–1.9))	0.33			
Plasma cells	1	(0.17–5.9)	1			
Neutrophils	0.25	(0.03–1.9)	0.18			
Eosinophils						
Fibrosis	1	0.38–2.6))	1			
Focal	Ref	Ref				
Dense						

VF = Vocal FoldCI = Confidence Interval* Significant level of p- value is < 0.05

In univariate regression analysis for factors predicting improvement among cases, significant predictors associated with improvement were not affected pharynx, absence of dysphonia, normal VF color, normal VF mucosa, no supraglottic or subglottic affection, treatment with levofloxacin. However, in multivariate regression, the only two significant were not affected pharynx and type of treatment. Treatment with levofloxacin was associated with higher odds (5.8) for improvement, *P* = 0.015 (Table 6).

## Discussion

Scleroma is a chronic granulomatous infection affecting the upper respiratory tract, predominantly involving the nasal cavity and is caused by *Klebsiella rhinoscleromatis*, a member of the Enterobacteriaceae family^17^, the nasopharynx, paranasal sinuses, and larynx are the next most frequent locations^18,19^.

Clinical, histologic, and microbiological correlations are necessary for diagnosing upper respiratory tract scleroma. Lesions first appear at the nasal vestibule’s mucocutaneous junction before moving to the nasopharynx. The next location of affection is the subglottic epithelial transition^20^.

The mainstay of treatment is long-term antibiotic medication that targets intracellular bacteria for three to six months^19^. Second- or third-generation cephalosporins, fluoroquinolones, ampicillin, cotrimoxazole, tetracyclines, and streptomycin can all be employed^21^.

In the present study, most patients with scleroma were females (90%) and housewives (73.3%). This female predominance may be explained by sociocultural and environmental factors, as women, particularly housewives, are often more exposed to indoor air pollution and poor hygienic conditions^22^. Hormonal influences and differences in immune response might also play a role. Similar findings were reported by several studies in endemic regions, which also documented a higher prevalence among females, particularly in developing countries^10,23,24^. However, several previous studies reported no significant gender differences among patients with RS^25,26^. This discrepancy across studies may be attributed to variations in demographic characteristics, sample size, or study design.

The age of patients included in this current study ranged from (18 to 52 years) with a mean age of 36.3 ± 10.4 years, indicating that RS mainly affects adults in the middle age group.

This wide age range could be attributed to the chronic course of the disease, where early subclinical infection gradually manifests with age. In addition, middle-aged adults usually represent the most active segment of the population, with higher levels of environmental exposure, which may increase the likelihood of infection or reactivation. Our findings agreed with previous studies that reported a higher prevalence of RS among middle-aged individuals^10,26,27^. Also agreed with *Fattah et al.*^28^, , who reported 48 scleroma patients, their age ranged from (17 to 60 years) with a mean of 39.04 ± 10.5 years, *Bakhiet et al.*^13^, , who reported 20 scleroma patients, their age ranged from (18to 50years) with a mean age of 30.98 ± 9.02 years, and *Awad et al.*^24^, , who reported 300 patients their age ranged from 19 to 60 years, with a mean age of about 37 years.

The limited number of smokers in our cohort (6.7%) suggests that smoking may not be a major contributing factor in disease development. The relatively low prevalence of chronic comorbidities, such as diabetes mellitus and hypertension, may be partially explained by the inclusion of participants aged 18–60 years only, as the prevalence of these conditions generally increases with advancing age. Therefore, the role of systemic illness in the pathogenesis of RS could not be adequately assessed in the present study.

In the current study, the most frequent clinical manifestations were nasal obstruction and crustations, followed by voice-related symptoms and postnasal discharge. These findings reflect the chronic granulomatous nature of the disease, while nasal mucosal thickening and crust formation lead to progressive nasal obstruction. The high percentage of voice change and phonasthenic symptoms could be attributed to the extension of the disease to the laryngeal structures in a considerable proportion of cases. Our findings are consistent with previous studies that reported similar clinical patterns^8,24,29^.

In the present series, nasal mucosa was affected in 100% of cases which is consistent with the literature describing the nasal cavity as the primary site of involvement in scleroma. *De Champs et al.*^30^, reported that the disease typically begins in the nasal mucosa and progresses from a catarrhal inflammatory phase to a granulomatous proliferative phase and finally to a sclerotic stage characterized by fibrosis and airway narrowing^13,31^.

Laryngeal involvement was observed in 86.7% of cases, which is higher than most previously reported series. As reported by *Abalkhail et al.*^5^, , the incidence of laryngeal involvement was 44%, while *Bakhiet et al.*^13^, found 10% in their cohort. Another study by *Awad et al.*^24^, , demonstrated an incidence of 60%. This higher incidence of laryngeal involvement in our series may be attributed to the endemic nature of scleroma in our region, delayed patient presentation, the chronic course of the disease, and referral bias, as our center is a tertiary referral hospital that receives more advanced and complicated cases.

Our findings showed that the subglottic region was the most affected part of the larynx showing greenish crustations and granulation, which is consistent with the literature reporting it as the most commonly involved site^21,24^.

Histopathological examination of upper respiratory scleroma cases in the current study revealed that the most common type of covering epithelium was squamous (80%), followed by columnar and the shedded epithelium (10% each). The inflammatory reaction was predominantly dense (66.7%), indicating a chronic inflammatory process. Russell bodies were detected in 66.7% of cases, reflecting the long-standing plasma cell response. Plasma cells were the dominant inflammatory cells (60%), followed by neutrophils and lymphocytes. Fibrosis was observed in all cases, with dense fibrosis in the majority (66.7%), suggesting a chronic and advanced stage of the disease.

In a retrospective study by Ahmed et al. involving 53 cases of rhinoscleroma, the epithelial covering was mainly of the squamous type in 88.7% of cases, while columnar and shedded epithelium were observed in 3.8% and 7.5% of cases, respectively. Russell bodies were identified in 66% of the specimens, and plasma cell infiltration predominated in 37.6% of cases. Fibrosis was reported in 77.7% of the cases^3^.

In this study, scleroma patients who received levofloxacin showed a higher rate of clinical improvement (80%) compared to those treated with third-generation cephalosporins (60%). This finding suggests that levofloxacin may provide better short-term clinical outcomes than third-generation cephalosporins in the management of upper respiratory tract scleroma.

The better outcome associated with levofloxacin could be related to its excellent tissue penetration and intracellular activity, which make it particularly effective against *Klebsiella rhinoscleromatis*, a facultative intracellular organism. Its concentration-dependent bactericidal effect and the prolonged tissue half-life may further explain the higher improvement rates observed in our cases.

Consistent with our findings, previous studies have also reported higher success rates with fluoroquinolones in treating patients with RS^13,32,33^. Previous case reports have shown that although third-generation cephalosporins are effective in the treatment of rhinoscleroma, relapse may occur following treatment discontinuation. *Trautmann et al.*^34^, reported bacteriological and/or clinical relapse in two of three patients treated with ceforanide despite its excellent in vitro activity against *Klebsiella rhinoscleromatis*. These findings are consistent with our results, which showed better short-term clinical outcomes with levofloxacin than with third-generation cephalosporins.

The current study identified several factors associated with better clinical outcomes in scleroma cases. Improvement was mainly related to the absence of pharyngeal or laryngeal involvement, as well as treatment with levofloxacin. These findings suggest that less extensive disease and preserved laryngeal integrity are favorable prognostic indicators. However, when adjusted for potential confounders in multivariate analysis, only pharyngeal affection and type of treatment remained significant predictors. This suggests that disease extension and antibiotic choice may be important determinants of response to therapy, with levofloxacin showing a higher likelihood of improvement.

Analysis of follow-up data revealed that patients treated with levofloxacin achieved better clinical improvement in most manifestations compared with those treated with third-generation cephalosporins. This was particularly evident in nasal obstruction, crustation, greenish expectoration, olfactory function, and vocal fold appearance. These findings suggest that levofloxacin may provide greater short-term improvement in several clinical manifestations, particularly those related to nasal and laryngeal involvement. However, longer follow-up studies are needed to confirm the durability of these outcomes.

As this was a single-center study conducted in a highly endemic region, the generalizability of the present findings to other populations and clinical settings should be interpreted with caution. Further multicenter studies involving more diverse populations are recommended to validate these findings.

## Conclusion

Levofloxacin was associated with higher short-term clinical improvement than third-generation cephalosporins in patients with upper respiratory tract scleroma during the three-month follow-up period. Better outcomes were associated with less extensive disease. These findings highlight the importance of early diagnosis and appropriate antibiotic selection in the management of upper respiratory tract scleroma. Further studies with larger sample sizes and longer follow-up are recommended to confirm the long-term efficacy of these treatment approaches.

### Limitations

The main limitation of this study was the relatively short follow-up period of three months, which might not be sufficient to assess the long-term recurrence of the disease. Extending the follow-up duration in future studies could provide a more comprehensive understanding of treatment sustainability and relapse rates. 

## Supplementary Information

Below is the link to the electronic supplementary material.

Supplementary Material 1

## Data Availability

Data availability statement: The datasets used and/or analysed during the current study are available from the corresponding author on reasonable request.

## Code availability

No software application or custom code was used in the study.
