# Supplementary material for: Comparative effectiveness of levofloxacin versus third-generation cephalosporins and predictors of treatment outcomes in upper respiratory tract scleroma: a tertiary center experience
Source: Sci Rep. 2026 Aug 20;16:26186. doi: 10.1038/s41598-026-66683-y (PMC13493878; doi:10.1038/s41598-026-66683-y)
Supplement: Supplementary file 1 [file 41598_2026_66683_MOESM1_ESM.docx]

**Predictors of Outcome of Levofloxacin Versus Third-Generation Cephalosporins in Treatment of Upper Respiratory Tract Scleroma: A Tertiary Center Experience**

**AUTHORS:**

1. **Ahmed Ibrahim Khaleel (corresponding Author)**

Lecturer at Otorhinolaryngology Department, Faculty of Medicine, Minia university, Minia, Egypt.

Email: [ahmed.khaleel@mu.edu.eg](mailto:ahmed.khaleel@mu.edu.eg)

ORCID: https://orcid.org/ 0000-0002-3243-0756

Telephone No.: +20101 4708388

2. **Rabeh Khairy Saleh**

Lecturer at Pathology Department, Faculty of Medicine, Minia University, Minia 61657, Egypt.

Email: [Rabeh.Saleh@mu.edu.eg](mailto:Rabeh.Saleh@mu.edu.eg)

ORCID: **0009-0007-6979-838X**

<https://orcid.org/0009-0007-6979-838X>

Telephone No.: +201220065443

3. **Chrestina Monir Fekry**

Lecturer at Public health and preventive medicine Department, Faculty of Medicine, Minia University, Minia, Egypt.

Email: [Christina.Minor@mu.edu.eg](mailto:Christina.Minor@mu.edu.eg)

4. **Fatma Wafik Shahin**

Unit of Phoniatrics, Otorhinolaryngology Department, Faculty of Medicine, Minia University, Minia, Egypt.

Email: [drfatmawafik@gmail.com](mailto:drfatmawafik@gmail.com)

ORCID: <https://orcid.org/0000-0002-5517-0432>

Telephone No.: +201004577996

**Acknowledgements:** Not applicable.

**Author contributions:** F.S. and A.K. conceived of the presented idea. F.S performed the endoscopic examination. A.K. performed biopsy for cases. C.F. developed the theory and performed the computations. C.F. and R.S. verified the analytical methods. R.S. performed the histopathological tests for the research participants. All authors discussed the results and contributed to the final manuscript.

**Declarations:**

**Ethical Approval**

The study had local Research Ethics approval of Minia medical collage ethics committee (approval number 1272/9/2024), in accordance with the ethical principles outlined in the Declaration of Helsinki, and written consent was obtained from patients.

**Competing interests:** The authors declare no conflict of interest.

**Funding:** No sources for fund

No financial support or other benefits from commercial sources for the work reported on in the manuscript, No other financial interests of the authors.
